# Peanut oral immunotherapy may improve health‐related quality of life among severe peanut allergic adolescents

**DOI:** 10.1002/clt2.12225

**Published:** 2023-02-05

**Authors:** Maria Ödling, Ann‐Charlotte Sundqvist, Josef Brandström, Anna Nopp, Niklas Andersson, Caroline Nilsson, Inger Kull

**Affiliations:** ^1^ Department of Clinical Science and Education Södersjukhuset Karolinska Institutet Stockholm Sweden; ^2^ Sachs' Children and Youth Hospital Stockholm Sweden; ^3^ Astrid Lindgren Children's Hospital Karolinska University Hospital Stockholm Sweden; ^4^ Clinical Epidemiology Division Department of Medicine, Solna, Karolinska Institutet Stockholm Sweden; ^5^ Institute of Environmental Medicine Karolinska Institutet Stockholm Sweden

To the Editor,

Peanut allergy is one of the major food allergies, and has an impact on the health‐related quality of life (HRQoL).^1^ As for today there is no curative treatment, however guidelines recommend oral immunotherapy (OIT) as a therapeutic option to increase the threshold for reactions in children with persistent peanut allergy.^2^, ^3^ In line with the guidelines, measurements of treatment effect should include patient‐reported outcomes such as HRQoL. Our aim was therefore to investigate if treatment with peanut OIT (pOIT) in combination with omalizumab improves self‐reported HRQoL among adolescents with severe peanut allergy.

The study is a part of a one‐armed open phase‐two study of pOIT in combination with omalizumab in severely peanut allergic adolescents (*N* = 23) (Figure 1, supplement 1).^4^ The patients received omalizumab for 8–24 weeks until vitro Basophil activation (CD‐sens) to peanut stimulation was suppressed. To measure HRQoL, the food allergy questionnaire Food Allergy Quality of Life Questionnaire–Teenager Form (FAQLQ‐TF) was used.

**FIGURE 1:**
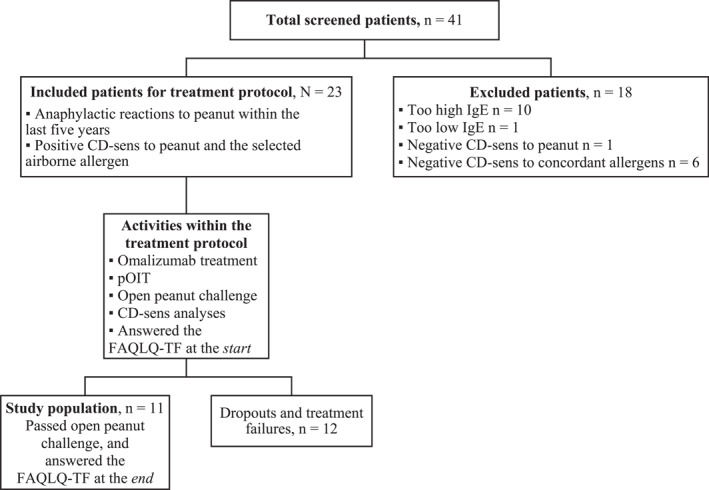
Flow chart of the study design and study population (*n* = 11).

The study population consisted of 11 (of the 23 included) patients who were able to continue with pOIT after the omalizumab treatment was stopped, and who completed the FAQLQ‐TF at the start and the end of the study (Figure 1). The study participants had a mean age of 15.8 years, and the mean length of treatment with omalizumab was 93 weeks (range 46–163). The distribution of sex and co‐morbidity were similar in the study population and among dropouts and treatment failures that is, participants who could not complete the treatment within scheduled time (*n* = 12) (supplement 2).

The FAQLQ‐TF mean scores significantly decreased at the end of the treatment in all domains (“Allergen avoidance and dietary restrictions”, “Emotional impact”, “Risk of accidental exposure”), and in the total score (Figure 2). To distinguish the clinical relevance of a difference, clinical minimal important difference (MID) is often used. A clinical relevance is suggested at a difference of ≥0.5 points.^5^ The MID at the end of the treatment in “Allergen avoidance and dietary restrictions” was 1.7 points lower than at the start, “Emotional impact” 1.6, and “Risk of accidental exposure” 1.4. In the total score, MID at the end of the treatment was 1.7.

**FIGURE 2:**
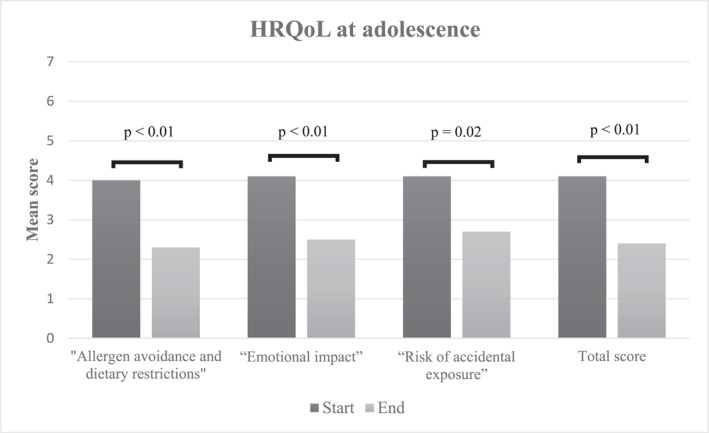
HRQoL among the study population (*n* = 11) at the start and at the end of the study.

In adolescence, HRQoL after OIT is sparsely studied. In the present study, HRQoL improved in all domains after OIT. For the “allergen avoidance and dietary restrictions” it is interesting, since adolescence is a period in life when the individuals spend more time outside the home and need to take responsibility of making food‐related decisions themselves compared to when they received help from their guardians. For the improvement in the “emotional impact”, one could speculate that after a long treatment with OIT and getting older, the adolescents got to know their disease, and feel more mature in case of an allergic reaction. Moreover, for “risk of accidental exposure” the result may reflect the increased tolerance to peanuts due to OIT, where the adolescents to a greater extent feel that they may be part of social events and even be more liberal in their diet. However, OIT is a slow and burdensome process and may need to be continued indefinitely. Therefore, benefits and downsides related to the treatment should be based on shared decision making together with the patient.^6^

The strength of the study includes the well characterised study population of highly sensitized peanut allergic patients with a history of anaphylactic reactions. Further, the FAQLQ‐TF is valid and reliable. The main limitations are small sample size and lack of control subjects.

In conclusion, the severe peanut allergic adolescents self‐reported an improved HRQoL after combined treatment with pOIT and omalizumab, and the difference from start were clinically relevant.

## AUTHOR CONTRIBUTIONS

Maria Ödling and Niklas Andersson have done the statistical analyses. Maria Ödling has written the manuscript, and together with Inger Kull planned this investigation. Ann‐Charlotte Sundqvist and Josef Brandström have collected the data and contributed to study design and information on the procedures. Anna Nopp and Caroline Nilsson have worked with the design of the study. All co‐authors have contributed with scientific knowledge, and read and critically revised the manuscript.

## CONFLICT OF INTEREST STATEMENT

The author declares no conflicts of interest.

## FUNDING INFORMATION

Torsten Söderbergs’s Foundation, the Swedish Asthma and Allergy Association, Mjölkdroppen Foundation, Hesselman’s Foundation, the Swedish order of Freemasons, Her Royal Highness Crown Princess Lovisa’s research fund, Region Stockholm (ALF‐project), Konsul Th C Bergh’s Foundation, The Swedish Association for Allergology, and Sachs’s Children and Youth Hospital.

## Supporting information

Supplementary Material

Supplementary Material
