# Supplementary material for: Peanut oral immunotherapy may improve health‐related quality of life among severe peanut allergic adolescents
Source: Clin Transl Allergy. 2023 Feb 5;13(2):e12225. doi: 10.1002/clt2.12225 (PMC9899491; doi:10.1002/clt2.12225)
Supplement: Supplementary file 1 — Supplementary Material [file CLT2-13-e12225-s001.docx]

**Supplement 1 - detailed method**

**Methods**

***Study design***

Severely peanut allergic adolescents from the FASTX (Food Allergen Suppression Therapy with Xolair ®) study previously described were included in the study (1-3). In brief, 23 peanut-allergic adolescents with documented anaphylactic reactions to peanut and who all were positive in peanut-specific basophils activation tests (CD-sens) were included in the study. All adolescents (N = 23) started oral immunotherapy (OIT) with 280 mg of peanut protein after they went through a peanut challenge. The peanut OIT (pOIT) dose was increased up to a maintenance dose of 2800 mg in 8 weeks followed by an individualized stepwise withdrawal of omalizumab, based on clinical symptoms and CD‐sens levels. POIT continued for 12 weeks after omalizumab withdrawal followed by an open peanut challenge and CD-sens analysis.

***Assessment of health-related quality of life***

Health-related quality of life (HRQoL) was measured using the Swedish version of EuroPrevall’s disease-specific food allergy HRQoL questionnaire-teenager form (FAQLQ-TF), at the start and the end of the study (4, 5). The FAQLQ-TF questionnaire is reliable, valid, short, and easy to use and is therefore a useful tool in clinical research. FAQLQ-TF consists of 28 questions on HRQoL, each of which has closed-ended answers on a 7-point scale where 1 is best and 7 is worst. The questions are also designed to address three domains: “allergen avoidance and dietary restrictions”, “emotional impact”, and “risk of accidental exposure”.

***Statistical analyses***

Descriptive data are presented as numbers (n) and percentages (%) for categorical variables (sex and co-morbidity), and as medians, ranges and means for continuous variables (IgE, IgG, FAQLQ-TF). Fisher’s exact test and T-tests were used to study differences between groups, and a p-value of < 0.05 was considered statistically significant. The total FAQLQ-TF score is based on the average score of all individual questions. To calculate the domain score, the sum of all question scores in the domain were divided with the number of completed questions in the domain. Analyses were performed with STATA Statistical Software (release 14.0; College Station, TX).

**Ethical approval**

The study was approved by the ethics committee in Stockholm: 2013/827-31/3, 2014/1980-32, 2016/1390-32, 2020-00807 and the Swedish Drug Agency: 5.1-2013-46183; The trial is registered at EudraCT: 2012-005625-78, ClinicalTrails.gov; NCT02402231. Patients and caregivers provided written informed consent.

**References**

1. Brandström J, Vetander M, Lilja G, Johansson SG, Sundqvist AC, Kalm F, et al. Individually dosed omalizumab: an effective treatment for severe peanut allergy. Clinical and experimental allergy : journal of the British Society for Allergy and Clinical Immunology. 2017;47(4):540-50.

2. Brandström J, Vetander M, Sundqvist AC, Lilja G, Johansson SGO, Melén E, et al. Individually dosed omalizumab facilitates peanut oral immunotherapy in peanut allergic adolescents. Clinical and experimental allergy : journal of the British Society for Allergy and Clinical Immunology. 2019;49(10):1328-41.

3. van der Heiden M, Nopp A, Brandström J, Carvalho-Queiroz C, Nilsson C, Sverremark-Ekström E. A pilot study towards the immunological effects of omalizumab treatment used to facilitate oral immunotherapy in peanut-allergic adolescents. Scandinavian journal of immunology. 2021;93(4):e13005.

4. Flokstra-de Blok BM, DunnGalvin A, Vlieg-Boerstra BJ, Oude Elberink JN, Duiverman EJ, Hourihane JO, et al. Development and validation of the self-administered Food Allergy Quality of Life Questionnaire for adolescents. The Journal of allergy and clinical immunology. 2008;122(1):139-44, 44.e1-2.

5. Protudjer JL, Jansson SA, Middelveld R, Östblom E, Dahlén SE, Arnlind MH, et al. Impaired health-related quality of life in adolescents with allergy to staple foods. Clinical and translational allergy. 2016;6:37.
