# Supplementary material for: Peanut oral immunotherapy may improve health‐related quality of life among severe peanut allergic adolescents
Source: Clin Transl Allergy. 2023 Feb 5;13(2):e12225. doi: 10.1002/clt2.12225 (PMC9899491; doi:10.1002/clt2.12225)
Supplement: Supplementary file 2 — Supplementary Material [file CLT2-13-e12225-s002.docx]

**Supplement 2**

**Table 1**. Distribution of patient characteristics among the study population (n = 11), and dropouts and treatment failures (n = 12).

| ***Patient characteristics*** | **Study population**  n = 11 | | **Dropouts and treatment failures**  n = 12 | |  |
| --- | --- | --- | --- | --- | --- |
|  | n | % | n | % | p-value^ⴕ^ |
| **Sex**, female | 9 | 81.2 | 7 | 58.3 | 0.37 |
|  |  |  |  |  |  |
| **Co-morbidity** |  |  |  |  |  |
| Doctors diagnosed asthma | 10 | 90.9 | 10 | 83.3 | 1.00 |
|  |  |  |  |  |  |
| Doctors diagnosed allergic rhinitis | 8 | 72.7 | 12 | 100.0 | 0.09 |
|  |  |  |  |  |  |
| Doctors diagnosed eczema | 3 | 27.3 | 3 | 25.0 | 1.00 |
|  |  |  |  |  |  |
| Allergy to furry pets | 10 | 90.9 | 8 | 66.7 | 0.32 |
|  |  |  |  |  |  |
| Allergy to pollen | 8 | 72.7 | 12 | 100.0 | 0.09 |
|  |  |  |  |  |  |
|  | Median | Range | Median | Range | p-value^ⱡ^ |
| **Total IgE**, kU/L |  |  |  |  |  |
| Start | 436 | 106–946 | 557 | 293–1026 | 0.45 |
| End | 560 | 190–1300 | NA | NA | - |
|  |  |  |  |  |  |
| **IgE-ab**, kU_A_/L |  |  |  |  |  |
| *Peanut* |  |  |  |  |  |
| Start | 52.0 | 30.0–260 | 200 | 32.0–350 | 0.04 |
| End | 81.0 | 37.0–420 | NA | NA | - |
|  |  |  |  |  |  |
| *Ara h 2* |  |  |  |  |  |
| Start | 30.0 | 16.0–102 | 63.5 | 24.0–220 | 0.05 |
| End | 60.0 | 20.0–94.0 | NA | NA | - |
|  |  |  |  |  |  |
| **IgG-ab** |  |  |  |  |  |
| *Peanut* |  |  |  |  |  |
| Start | 8.5 | 4.6–21.9 | 12.7 | 5.4–21.6 | 0.23 |
| End | 47.6 | 11.6–708 | NA | NA | - |
|  |  |  |  |  |  |
| *Ara h 2* |  |  |  |  |  |
| Start | 6.7 | 2.0–27.4 | 6.8 | 4.5–16.1 | 0.70 |
| End | 42.6 | 11.6–177 | NA | NA | - |
|  |  |  |  |  |  |
| **IgG4-ab** |  |  |  |  |  |
| *Peanut* |  |  |  |  |  |
| Start | 0.38 | 0.02–2.8 | 0.43 | 0.23–0.94 | 0.64 |
| End | 26.0 | 1.6–493 | NA | NA | - |
|  |  |  |  |  |  |
| *Ara h 2* |  |  |  |  |  |
| Start | 0.11 | 0.01–0.36 | 0.28 | 0.10–0.56 | 0.01 |
| End | 29.2 | 1.9–223 | NA | NA | - |

^ⴕ^P-values indicated differences between the study population and dropouts and treatment failures, obtained using Fisher’s exact test.

^ⱡ^P-values indicated differences between the study population and dropouts and treatment failures, obtained using T-test.
